# Fund behavioral science like the frameworks we endorse: the case for increased funding of preliminary studies by the National Institutes of Health

**DOI:** 10.1186/s40814-022-01179-w

**Published:** 2022-09-28

**Authors:** Michael W. Beets, Christopher Pfledderer, Lauren von Klinggraeff, Sarah Burkart, Bridget Armstrong

**Affiliations:** Arnold School of Public Health, University of South Carolina, Columbia, SC USA

**Keywords:** Feasibility, Translational Science, Pilot, Framework, Scaling, Innovation

## Abstract

Innovative, groundbreaking science relies upon preliminary studies (aka pilot, feasibility, proof-of-concept). In the behavioral sciences, almost every large-scale intervention is supported by a series of one or more rigorously conducted preliminary studies. The importance of preliminary studies was established by the National Institutes of Health (NIH) in 2014/2015 in two translational science frameworks (NIH Stage and ORBIT models). These frameworks outline the essential role preliminary studies play in developing the next generation of evidence-based behavioral prevention and treatment interventions. Data produced from preliminary studies are essential to secure funding from the NIH’s most widely used grant mechanism for large-scale clinical trials, namely the R01. Yet, despite their unquestionable importance, the resources available for behavioral scientists to conduct rigorous preliminary studies are limited. In this commentary, we discuss ways the existing funding structure at the NIH, despite its clear reliance upon high-quality preliminary studies, inadvertently discourages and disincentivizes their pursuit by systematically underfunding them. We outline how multiple complementary and pragmatic steps via a small reinvestment of funds from larger trials could result in a large increase in funding for smaller preliminary studies. We make the case such a reinvestment has the potential to increase innovative science, increase the number of investigators currently funded, and would yield lasting benefits for behavioral science and scientists alike.

**Supplementary Information:**

The online version contains supplementary material available at 10.1186/s40814-022-01179-w.

“It is impossible to get large behavioral trials funded without conducting preliminary studies... and there is also very limited federal funding available for critical preliminary studies … this has made it much harder for young behavioral scientists to establish themselves and for established behavioral scientists to continue to pursue developing novel behavioral interventions.” -Anonymous female behavioral scientist with 24 years’ experience^1^

The origin of almost every biomedical and behavioral science breakthrough can be traced to discoveries emerging from one or more preliminary studies [1–12]. For the purpose of this commentary, we use the term preliminary studies^2^ to refer to those studies conducted at the early stages of intervention conceptualization, development, testing, and refinement which are designed to assess the feasibility (can we do it) and potential impact (could it work) of an intervention. This information is used to make decisions about whether an intervention is ready to be tested in a larger-scale, more well-powered clinical trial.

Innovative, groundbreaking science requires preliminary studies and preliminary studies are the testing ground for innovative ideas. While a large proportion of innovations ‘fail’ [13, 14], ideas that demonstrate initial promise in preliminary studies serve as the catalyst for major breakthroughs in future prevention and treatment interventions. Without preliminary studies to conduct “risky” and innovative science, many effective prevention and treatment interventions might never be discovered.

Developing innovative behavioral interventions requires conducting multiple iterations of an intervention to refine, modify, and/or adapt its content to identify the maximally potent prevention or treatment package [4, 15]. If designed, executed, and interpreted appropriately, preliminary studies can identify potential weaknesses early in the lifecycle of an intervention and can help avoid failures in larger-scale trials [16–18]. Failing and discovering on a smaller scale, on a more abbreviated timeline, accelerates the pace for discovering more efficient, effective, and enduring behavior interventions [19].

Given their importance, resources to conduct preliminary studies should be abundant. Unfortunately, this is not the case. In this commentary we discuss ways the existing funding structure at the National Institutes of Health (NIH), despite its clear reliance upon “strong”, “solid”, and “high-quality” preliminary studies [20], substantially underfunds preliminary studies which inadvertently discourages and disincentivizes their pursuit. We focus our attention on the role the NIH, as the largest funder of behavioral sciences in the USA and the largest agency in the world that funds health research [21], plays in funding preliminary studies. We describe how a pragmatic reallocation (i.e., reinvestment) of their current portfolio could dramatically increase the number (and quality) of preliminary studies conducted. We make the case that a small reinvestment of funds towards preliminary studies has the potential to yield large, immediate, and lasting benefits on behavioral science and scientists alike.

This commentary begins with a discussion on the importance and recent emphasis the NIH has placed on conducting high-quality preliminary studies to inform larger-scale trials and is followed by a historical accounting of the funds allocated towards preliminary studies by the NIH. These sections are followed by a description of six complementary and pragmatic strategies to expand funding for preliminary studies through reinvesting funds from larger-scale trials. The commentary ends with a discussion about who may benefit from such reinvestment of funding from a career, science, and societal impact.

To illustrate and emphasize these points throughout, we use quotes from an open-ended survey of over 400 NIH-funded Principal Investigators about the importance of preliminary studies. Briefly, a survey was sent to Principal Investigators funded by the NIH to conduct a preliminary study on a behavioral intervention. One section of the survey allowed for open-ended free-text responses regarding challenges in the field around the importance, design, conduct, and funding of preliminary studies. The quotes presented represent a sample from those the open-ended responses. Additional details can be found in Additional file 1: Methods for qualitative quotes at the end of this commentary.

## The NIH and the importance of preliminary studies

“They [preliminary studies] are the cornerstone to the development of the larger scale studies.” -Anonymous female behavioral scientist with 33 years’ experience

“Preliminary studies are often seen as an essential step (and expected in order to obtain funding).” -Anonymous female behavioral scientist with 3 years’ experience

**Fig. 1:**
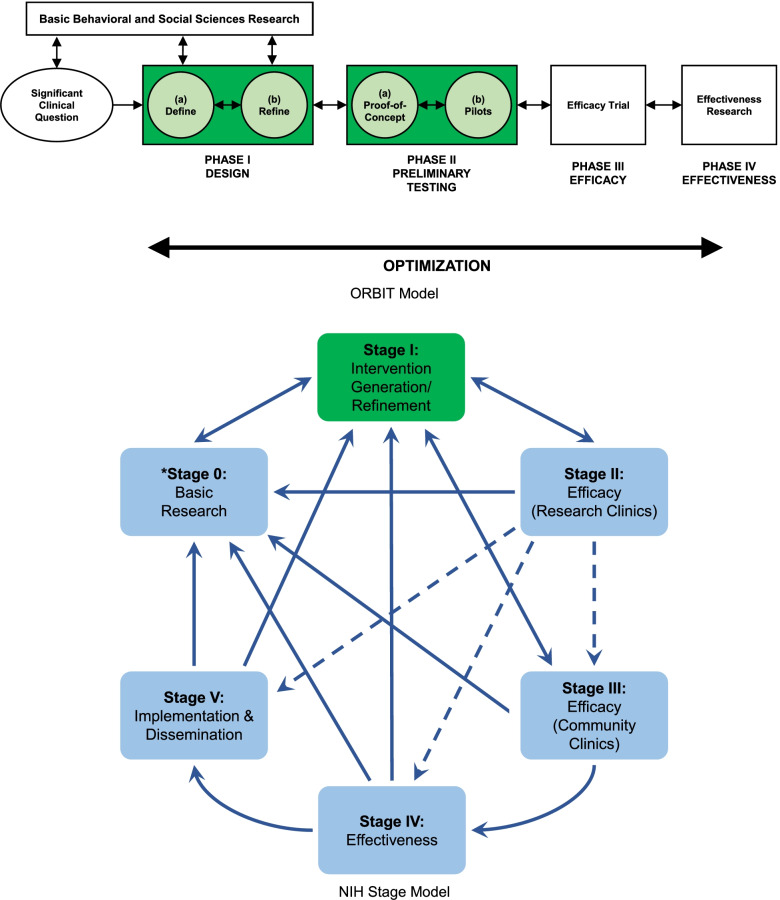
Behavioral science intervention development frameworks. Preliminary studies phases (ORBIT) or stages (NIH Stage Model) are depicted in green

The NIH has long recognized the important role preliminary studies play in discovering and informing the development of future prevention and treatment interventions. In 2014 and 2015, several of the NIH’s largest Institutes published two frameworks—the NIH Stage Model [15] and the Obesity-Related Behavioral Intervention Trials (ORBIT) Model [4]—which are designed to provide guidance to behavioral scientists on the development of maximally impactful evidence-based behavioral interventions. The frameworks outline sequential and iterative processes for creating and refining larger-scale behavioral interventions through the extensive testing (and retesting) of interventions in a series of preliminary studies (see Fig. 1). More recently, in the NIH-wide strategic plan for 2021–2025 [21], attention is focused on the importance of foundational science (synonymous with preliminary studies) to generate new knowledge to address key gaps in prevention and treatment science. The development and adoption of these frameworks and the emphasis on preliminary studies in the strategic plan has advanced how behavioral scientists approach developing and optimizing interventions. Importantly, these publications heightened the importance of conducting one or more high-quality, informative preliminary studies to justify the conduct of a larger-scale, more definitive trial which has become “industry standard” by behavioral scientists in the development of behavioral interventions.

Beyond providing evidence to support a larger trial, preliminary studies provide an opportunity for investigative teams to improve multiple facets of a study, which include refining protocols, documentation, and consent procedures. Each of these improvements has the potential to enhance the overall rigor and reproducibility of an intervention [22]. The importance of preliminary studies is conveyed in the explicit requirements for pursuing funding for larger-scale trials where “strong,” “solid,” and “high-quality” preliminary data can “make all the difference” [20] if an application is favorably reviewed and funded. Preliminary studies are so important that even mechanisms commonly used to propose preliminary studies (e.g., R21, R03), while not explicitly requiring preliminary data, rely heavily upon preliminary data to be favorably reviewed [20]. Thus, every large- (and small-) scale trial requires initial evidence about whether the research can be done (feasibility), whether it can potentially lead to improvements in health (impact), and whether the research is worth the money [23].

## Current NIH funding allocations for preliminary studies

“Preliminary data is required for an NIH R01 clinical trial...not having a convincing pilot trial is a non-starter. The bar is higher now than in the past … ”-Anonymous male behavioral scientist with 30 years’ experience

“They [preliminary studies] are no less work to get funded than full trials … ”-Anonymous female behavioral scientists with 11 years’ experience

The NIH has a variety of mechanisms to generate preliminary data as well as support larger-scale clinical trials. Our discussion here focuses on those mechanisms that are widely issued across NIH Institutes and are available to investigators across all career stages.^3^ For the purpose of this commentary, we draw attention to the R21, R03, R34, and K award mechanisms to support preliminary studies and the R01 to support larger-scale clinical trials. We recognize other mechanisms^4^ can support both preliminary studies and larger-scale trials. However, these are not available to investigators at all career stages or are sparsely used.

**Fig. 2:**
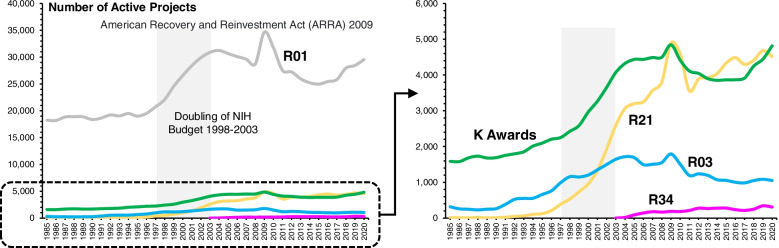
Total number of active project awards by mechanism from 1985 to 2020. Source: NIH Reporter

The NIH recognizes the usefulness of smaller grant mechanisms for the testing of innovative and pragmatic approaches to behavior change [24]. Most common in the NIH are the R21, R03, R34, and Research Career Award (K awards) mechanisms [25]. These awards last 2–5 years in duration and fund anywhere from $25,000 per year to $175,000 per year in direct costs [19]. As shown in Fig. 2, from 1985 to 1997, the NIH funded an average of 2965 preliminary studies (i.e., R21, R03, R34, and K awards). The number steadily increased to ~ 9500 per year by 2005 due to the doubling of the NIH’s budget from 1998 to 2003 (see Fig. 2). This increase equates to a growing investment in preliminary studies from an average of $174.8 million per year between 1985 and 1997 to an average of $1.69 billion per year since 2005. Since 2005, the average number of active preliminary studies has remained stable around 9855 (± 719) per year.

**Fig. 3:**
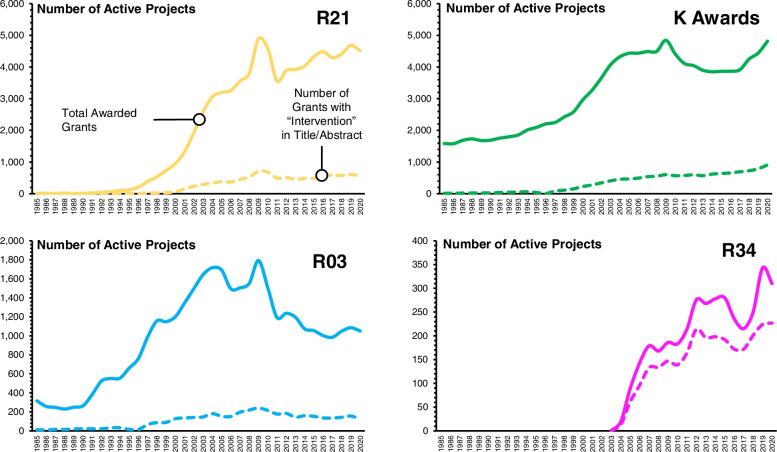
Comparison of total award projects be preliminary study mechanism and those preliminary studies with “intervention” in the title or abstract

Restricting the analysis to those studies with “intervention”^5^ in either the title or abstract reveals an even lower investment in preliminary studies for the behavioral sciences [26]. As shown in Fig. 3, from 1985 to 1997, the NIH funded an average of 5, 23, and 36 R21s, R03s, and K awards per year, which comprised 12%, 5%, and 2% of the total awards of each mechanism, respectively. This investment increased to an average of 529 R21s, 168 R03s, and 626 K awards per year from 2005 to 2020, with the proportion of interventions being 13%, 13%, and 15% respectively.

The most widely used NIH mechanism for funding larger-scale clinical trials is the R01. This is the NIH’s original, and oldest mechanism, and provides resources to conduct studies lasting 3–5 years (typically 4 or more) with direct costs up to $500,000 per year. As shown in Fig. 2, from 1985 to 1997 the NIH funded an average of 19,004 R01s each year or approximately 8 R01s for every 1 preliminary study (R21, R03, R34, or K Award). By 2005 the number of active R01s increased to over 30,000 annually. Since then, the NIH has funded an average of 28,350 R01s per year. This translates into approximately 3 R01s for every 1 preliminary study during that time.

**Fig. 4:**
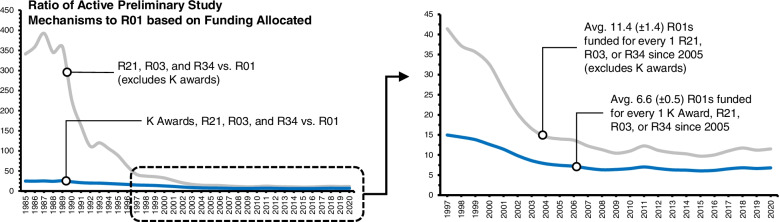
Ratio of funding between R01s and Preliminary Studies (R21, R03, R34, and K Awards) from 1985 to 2020. Source NIH Reporter

Because comparison of the number of grants awarded masks differences in the actual dollars invested, we also compared the number of active awards to the dollars invested over this same period between preliminary studies and R01s. In Fig. 4, we show the ratio of R01 funding to preliminary study funding from 1985 to 2020. Between 1985 to 1997 the NIH invested in an average of 19.8 R01s each year for every R21, R03, R34, or K Award. This ratio reduced and has remained stable since 2005 with an investment in 6.6 R01s each year for every R21, R03, R34, or K Award. Excluding K Awards,^6^ this ratio dramatically increases. Between 1985 and 1997 the NIH invested in 109 R01s each year for every R21, R03, or R34 and this declined and has remained stable at an average of 11.3 R01s for every R21, R03, or R34 since 2005.

## Reinvesting NIH funding for preliminary studies

“ … I love that they [NIH] have totally bought into focused intervention design, but it’s almost like that has just increased the expectations, without offering researchers a way to meet them.” -Anonymous female behavioral scientists with 27 years’ experience

“Behavioral trials are often expensive to conduct, so without funding for 1-2 preliminary trials, it can be very difficult to show efficacy that will interest reviewers in funding novel or innovative treatment strategies.” -Anonymous female behavioral scientist with 34 years’ experience

“We need more preliminary studies to avoid poor outcomes in large expensive RCTs.” -Anonymous female behavioral scientist with 37 years’ experience

Changing existing funding structures to invest in more preliminary studies is not an easy task [26, 27]. Research funding is finite [28] and, in absence of increases in federal budgets, funding more preliminary studies will require a reallocation of the funding for other types of studies. Below we present multiple complimentary and pragmatic strategies to expand funding for preliminary studies.

### Strategy 1: slightly reduce the number of R01s funded and reinvest in preliminary studies

**Fig. 5:**
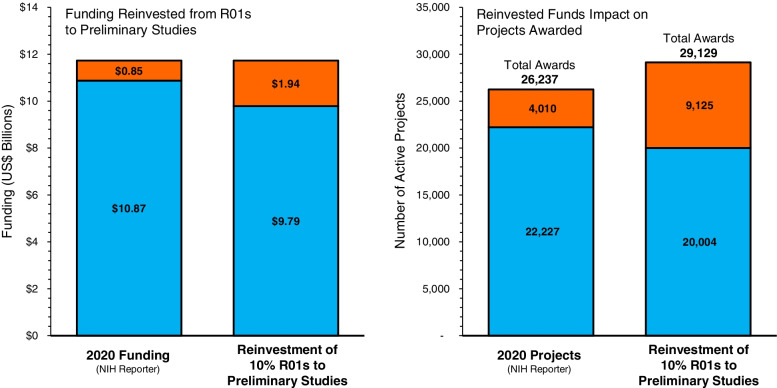
Example of reinvesting 10% of 2020 funding for R01s into preliminary studies (R21, R03, R34)*. Source NIH Reporter BLUE represents R01s; ORANGE represents preliminary studies (R21, R03, R34)*. *Note: K Awards are not included in these estimates given they are commonly awarded only once during an investigator’s career

Based on NIH Reporter data from 2020, a 10% reduction in R01s across the top 9 Institutes^7^ (22,227 to 20,004 projects) would free $1.09 billion/year (see Fig. 5). Over a 4-year span (estimating the average length of an R01) these monies could be reinvested to support an additional 10,229 new R21, R03, and R34 projects (estimating the average length of a preliminary study is 2 years)^8^. This would double the number of active R21, R03, and R34 preliminary study projects each year from 4010 to 9125 across the 9 institutes. If the 2223 R01s (10% reduction) were distributed evenly over the same number of PIs and any given PI could lead 2 active preliminary studies during a 4-year period, the reinvested funds could support a total of 5115 PIs over 4 years, resulting in an increase of an additional 2892 PIs receiving NIH funding. If we assume half of these projects would be led by an early-career researcher^9^, this will double the number of early-career researchers currently funded by the NIH’s Next Generation Researchers Initiative [21] from 1316 to 2762. Moreover, this reinvestment would result in an increase in the total number of R01, R21, R03, and R34 awards from 26,237 to 29,129 across these 9 Institutes.

### Strategy 2: raise paylines for awarding preliminary studies

The paylines for preliminary studies are either lower than the paylines for R01s (e.g., NCI 9th percentile for R21 vs 11th percentile for an R01) [29] or are not specified and left to an Institute’s discretion. As of December 2021, the overall success rate for receiving an R21 was lower than that for R01s [30]. It makes little sense that the ability to successfully compete for a substantially smaller amount of funding ($275,000 direct costs over 2 years) would be more difficult than competing for funding that is up to 9 times larger in direct costs over the life of the grant (R01 up to $2.5 million over 5 years). In conjunction with funding a larger number of applications, raising paylines for mechanisms like the R21 or R34 to the 25th percentile and the R03 to the 35th percentile would minimize the perception from behavioral scientists that pursing and receiving a smaller preliminary study’s grant is as difficult, if not more, than pursuing R01-level funding.

Raising the payline for preliminary studies is unlikely to reduce the quality of the studies funded. Analyses of over 100,000 funded NIH grants indicates percentiles based on peer review poorly discriminate productivity (as measured by publications/citations) for applications scored between the 3rd and 20th percentile [31]. Higher paylines would also align with how reviewers score preliminary study applications. As stated earlier, preliminary studies cannot guarantee success because they are testing innovative, novel ideas on a much smaller scale and are less adequately powered to detect changes in outcomes.

Each of these are weaknesses not commonly differentiated by reviewers when scoring preliminary studies yet are an inherent part of the development/refinement stage of the translational continuum for creating behavioral interventions [32]. When reviewers expect fully powered preliminary studies, they confuse the very purpose by which they exist—to inform decisions about whether an intervention is viable and holds promise for testing in a larger-scale trial. Thus, preliminary studies should not be held to the same standards as R01s, although the prevailing impression is that they commonly are. Previous studies show novel proposals receive worse scores by reviewers compared to proposals with low levels of novelty (i.e., less risky) [33]. When faced with a choice between unoriginal and creative ideas, reviewers show an implicit bias against creativity [34]. Creative ideas are more novel and a hallmark characteristic of preliminary studies. The greater the novelty the less certainty about an idea’s success. This is consistent with recent analyses indicating the types of research the NIH funds has become more conservative over time despite initiative and polices designed to increase innovativeness [35]. Educating reviewers regarding these nuances with preliminary studies may be a viable solution to improve peer review in the short term, but such efforts are unlikely to lead to long-term changes [36, 37]. Thus, raising paylines to accommodate how reviewers score preliminary studies should allow for more creative, novel, and innovative science to be awarded despite the inherent weaknesses attributed to them, such as the smaller sample size.

### Strategy 3: reinstate parent announcements for all preliminary studies mechanisms

Over the last two decades, several leading Institutes (e.g., NHLBI) have gone from accepting preliminary studies through broader, non-specific calls (i.e., parent announcements) to only accepting preliminary studies with more narrow scientific focus. Although it is recognized the best ideas come from investigators themselves, some Institutes have increased targeted, more narrowly defined scientific funding opportunities at the expense of investigator initiated (i.e., parent) research [38]. Concentrating funding opportunities into increasingly narrow scientific fields limits the diversity of scientific ideas and lowers the opportunity for emerging and possibly more promising areas of research to be funded [39]. These policies substantially narrow the options for investigators to propose new and innovative ideas that fall outside specific scientific foci but could be highly relevant to the Institutes. Thus, increased funding coupled with less restrictions on the types of ideas proposed should lead to a far greater number of innovative and potentially breakthrough ideas funded.

### Strategy 4: broadly use existing mechanisms across Institutes to support preliminary studies

The NIH has existing preliminary study mechanisms designed specifically for the development, testing, and refinement of an intervention. For instance, the NIH Phased Innovation Award (R21/R33) [40, 41], provides support for 2 years during the R21 phase to complete initial intervention development and feasibility-related studies, followed by 3 years to complete additional refinements and preliminary efficacy testing. An important aspect of these mechanisms are the milestones required to transition from the R21 to R33 phase. This is consistent with scientific advancements in defining progression criteria for conducting larger-scale trials [42]. Moreover, this expanded (up to 5 years) timeline provides opportunities to refine and retest an intervention based upon the outcomes from previous iterations. In 2020, only 264 R33 projects were active, indicating a very low use of this mechanism to generate preliminary evidence. Other institutes have retired preliminary study mechanisms designed specifically to generate data to support larger-scale clinical trials and replaced these with a single mechanism with a limited number of awarded applications [43]. The R34 is another mechanism designed to support preliminary studies. This award provides $450,000 in direct costs over 3 years. However, since its introduction in 2003, the NIH has awarded an average of 202 R34s per year with only slightly more per year since 2010 (259/year).

### Strategy 5: eliminate the use of the R01 mechanism, in name only, to fund preliminary studies

In recent years, multiple Institutes at the NIH issued funding opportunities using the R01 activity code to support the conduct of preliminary studies [44, 45]. The funding opportunities were issued in response to the hypercompetitive funding environment, designed to address the challenges early-stage investigators face in securing funding, and intended to generate the necessary preliminary data required to support the conduct of a larger clinical trial. While this is clearly a move in the right direction, there are issues with the use of the R01 activity code designation that potentially undermine these efforts. The major issue with these funding opportunities is they are R01 in name only and do not allow for the conduct of R01-eqivalent science. One opportunity is referred to as a “small” R01 and provides up to $200,000 per year in direct costs for only 3 years. The other lasts up to 5 years but caps the total direct costs at $250,000 per year.

Labeling preliminary studies as R01s creates false pretenses under which such studies get reviewed. During the review process, applications labeled as R01s trigger a different level of expectations by reviewers for the science proposed versus the review of other mechanisms, such as R21s and R03s. Scientific review officers provide detailed trainings to study section members regarding the differences among mechanisms to assist with an appropriate review of the science proposed. Study sections also review R01s separately from other mechanisms because of this very reason. The advent of these R01 in name only applications creates a scenario where they are comingled with “large” R01s ($500k/year up to 5 years) for review. This only serves to confuse reviewers further about how to appropriately evaluate the science proposed in “small” R01s designed to generate preliminary data (see “Strategy 2: raise paylines for awarding preliminary studies” section for details regarding review of preliminary studies).

Perhaps, the most unintended consequence of these opportunities is their impact on early-career investigators. The most important advantage early-stage investigators have in securing their first R01 is the differential payline at which their applications are evaluated for funding. The creation of the “small” R01 runs the risk of early-stage investigators using this “more generous” payline for an “R01” that is clearly smaller in scope compared to an actual R01 [46]. Thus, early-career investigators awarded a R01 via these opportunities will inadvertently give up their highly coveted early-stage investigator (ESI) status to do non-equivalent research labeled as an R01 in name only.

With the recognized difficulties in securing R01-leveling funding across all career stages, capping direct costs and shortening timeframes of an R01, yet retaining a label that infers a level of science in both scope and rigor that simply cannot be supported, only serves to disadvantage investigators from successfully receiving funds for preliminary studies and penalize those ESIs that are awarded.

### Strategy 6: retire the narrative that R21, R03, and R34 like mechanisms are insufficient to generate strong, solid, and high-quality preliminary data

The prevailing narrative regarding several of the most common, smaller mechanisms is they are not designed to generate preliminary data [47]. If 2 years and $275,000 is insufficient to conduct a preliminary study, exactly what is? The NIH points to other sources of funding for preliminary studies such as start-up funds, institutional grants, professional societies, and career development awards [20]. These sources are unlikely to provide resources above $275,000 in direct cost. Career awards, while longer in duration, come with very small amounts of funding for research activities ($25,000 to $50,000/year). Such a resource level is unlikely to support the generation of high-quality preliminary studies. Some institutes, however, have issued R21s with the explicit intention of generating preliminary studies [48] and to facilitate the transition to research independence (i.e., PI of an R01) [49]. The R21 mechanism is said to be intended for “exploratory/development” research characterized as high risk/high reward. If this doesn’t exemplify the very nature of preliminary studies, it is unclear what would. Thus, these mechanisms are indeed designed for collecting “solid,” “strong,” and “high-quality” preliminary data [20]. The reason we advise against their pursuit is because the current system makes it difficult to successfully pursue them through underfunding and overly critical reviews. The NIH and the behavioral science field needs to retire the narrative that some of the most popular mechanisms at the NIH are not designed to generate strong, solid, and high-quality preliminary studies when clearly, they are.

## Beneficiaries of reinvesting in preliminary studies

“The competition is just too fierce and it's exhausting … ” -Anonymous female behavioral scientist with 18 years’ experience

“Preliminary work is absolutely a place where inequities among institutions are solidified … ” -Anonymous female behavioral scientist with 13 years’ experience

“Preliminary studies and funding are extremely important to early-career investigators in behavioral interventions. They can potentially make or break our academic career.” -Anonymous female behavioral scientist with 8 years’ experience

### Beneficiaries early-career researchers

Early-career researchers often have less preliminary data than more established investigators [50]. Allocating more funding towards such studies would ease this issue. A greater number of opportunities to secure funding for preliminary studies would also alleviate new investigators from feeling “pressured” into pursuing an R01 immediately from a post-doctoral fellowship, an award size and research scope they may not feel adequately prepared to lead at that time [51]. Early-career researchers may feel R01s are their only option, given current funding allocations, or receive advice that pursuing funding for preliminary studies is more difficult than seeking R01-level funding [30], thereby reinforcing the idea they must pursue R01 funding. Advising early-career investigators to pursue R01 applications should not be justified simply because it is a larger amount of funding or longer funding period. More pragmatic reasons for advising which mechanism is the “right” one should be based on the strength of preliminary data, the stage at which research exists along the translational continuum, and whether the individual is ready to oversee an award of a given size. Bigger is not always better, regardless of career stage.

**Fig. 6:**
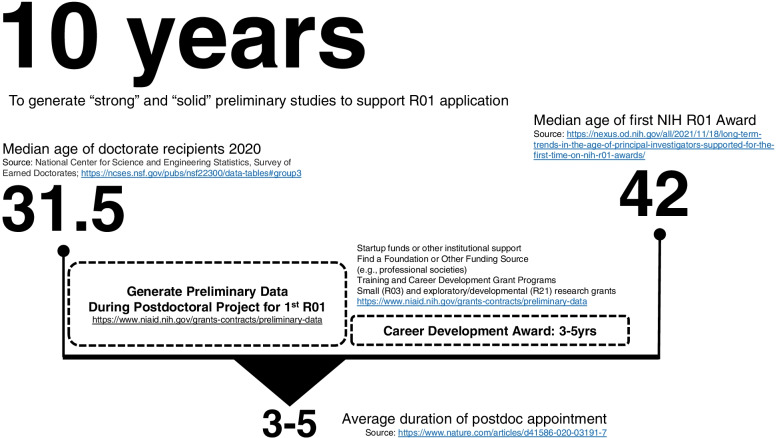
Timeline from graduation with terminal degree to receipt of first R01

Investing in more preliminary studies may also reduce the average age at which a PI receives their first successfully funded R01. Recent evidence indicates the median age is 42 years and this has increased over the past decades [52]. One reason for this may be a result of the struggle to generate enough strong, solid, and convincing preliminary data over the early part of one’s career. The median age at graduation from a doctoral program is 31 years [53]. Combine this with a 3–5 year post-doctoral fellowship [51] and the amount of time early-career investigators have after their terminal degree to receive their first R01 is over 10 years (see Fig. 6). Thus, the advice from the NIH that post-doctoral projects can provide evidence to support an R01 is inconsistent with these data [20]. With more funds invested in preliminary studies, early-career investigators may be able to shrink this sizeable timeline and accelerate their success towards their first R01.

Greater funding for preliminary studies would also help early-career researchers to persevere and pivot their research knowing there was more funding available to refine/retest existing ideas and to propose innovative and riskier new ideas. As early-career researchers depart from the work they conducted as a doctoral or post-doctoral fellow and begin to develop their independent area of research, greater funding of preliminary studies would assist them in generating the necessary evidence to branch out into new areas of science [54].

Pursuing funding for preliminary data may also be viewed as less intimidating for those newly entering the scientific workforce. Failing to successfully receive funding early in one’s career can diminish participation in future submissions, which may cause early-career researchers to leave their research field prematurely [39, 55]. Providing more funding for preliminary studies may improve the chances of early-career scientist successfully competing for research funding.

Providing more funding for preliminary studies has the potential to open the research scientist career path for those that may not traditionally be competitive for R01-level funding early in their careers [49]. Allowing more opportunities for individuals to develop strong research portfolios through preliminary studies could support scientists from lower-resourced institutions or from groups who have been historically underrepresented and may experience more challenges in obtaining the more traditional metrics of success during the early part of their career [56–59]. Funding a greater number of preliminary studies may prove to address the growing inequalities in the distribution of NIH funding across individuals and organizations [60]. Broadening the pool of talented and diverse research scientists, and as a natural byproduct, broadening the pool of innovate and untested ideas, should lead to a far greater number of breakthroughs in the behavioral sciences than funding a relatively smaller number of research scientists [14, 61].

Finally, funding a greater number of preliminary studies would provide much needed experience managing sizable extramural awards [62]. Proposing science is one part of a successful grant; the other is the daily management of the project, developing and overseeing the execution of procedures and protocols, and budgeting. Doing this well can be a steep learning curve, which should not come within the context of a 4–5 year multi-million-dollar project. Overseeing one or more smaller-sized preliminary studies could provide useful training/education to early career researchers about the overall processes of managing grants and prepare them to successfully manage multi-million-dollar awards.

### Beneficiaries mid-career and established researchers

Mid-career and established investigators would also benefit from expanded funding of preliminary studies [63] When pursing a novel idea or when taking existing interventions and adapting/modifying them for new contexts and populations, funds may not be readily available to support such efforts. Creating a larger pool of resources to pursue these for individuals across all career stages would allow for mid-career and established researchers looking to either expand their existing research or move into entirely new areas of research to propose significant, innovative, and risky scientific questions [54]. The NIH recognizes their preliminary study mechanisms are a useful and cost-effective way for investigators to enter into new areas of research [64] and past calls have been made for the expansion of funds for risk-taking endeavors [54]. Thus, expanding the amount of funds dedicated to preliminary studies would accelerate discoveries of potentially more enduring behavioral interventions [19]. The NIH also recognizes diversity in their funding portfolio, from the size of the grants to the stage of career of those funded, as a strength that should increase the probability of identifying the unexpected major discoveries [38]. It is impossible to know where the next breakthrough will come from and increasing funding for preliminary studies should accelerate these discoveries. A reinvestment in preliminary studies is likely to assist scientists across all career stages, not just those at the early part of their careers.

### Beneficiaries science and the public

“They [preliminary studies] are an indispensable part of intervention development. Our field progresses faster and is more impactful because of preliminary studies.” -Anonymous male behavioral scientist with 13 years’ experience

“There needs to be other funding mechanisms for preliminary studies - it would be a money-saving approach and potentially result in better long-term outcomes from behavioral studies.” -Anonymous female behavioral scientist with 43 years’ experience

In today’s hyper-competitive funding climate [65], it takes multiple submissions, often lasting several years, to successfully secure NIH funding. Knowing many investigators are unlikely to have multiple preliminary studies being conducted concurrently, added pressure may be present to make sure an existing preliminary study “pans out.” With limited preliminary study funding, non-financial sources of bias, such as careerism and confirmation bias, may influence interventionists to interpret findings from a single preliminary study as beneficial and go immediately for an R01. This can lead to funding larger-scale trials that contain one or more fatal flaws that are not sufficiently “ironed out” as they would through the conduct of two more preliminary studies.

Funding slightly fewer larger trials and funding substantially more preliminary studies should result in larger trials having a greater likelihood of success. This would benefit the scientific community and the general public by providing a greater opportunity for innovative ideas to be tested, those that fail to be “weeded out”, and the evidence upon which decisions to fund larger trials be better informed with high-quality preliminary data. More funding for preliminary studies would facilitate a greater use of innovative approaches, such as agile science [66] and iterative research designs [26], to develop the next generation of behavioral interventions. Investing in a greater number of preliminary studies allows for the cycle of development, failure, and refinement to occur continuously. Moreover, an expansion of the number of research scientists receiving funding would lead to a greater number of potentially groundbreaking ideas to be tested and refined. This is the very intent of the NIH Stage and ORBIT frameworks and the Next Generation Researchers Initiative.

Finally, the NIH stands to be one of the greatest beneficiaries of this reinvestment. Funding more scientists and identifying more novel and innovative research that could lead to major scientific breakthroughs at an accelerated rate are all issues the NIH has attempted to address over the past decades. Because the NIH is the largest actor in the research funding system, they have the greatest power to enact such changes to make an immediate and positive impact on behavioral scientists and science alike. In the past, when the NIH made funding changes, the system reacted in the direction of the reallocation [67]. Thus, the NIH cannot serve as a passive actor in the research system regarding these issues because they are the system. As the largest funder of behavioral science, there is an obligation to reevaluate existing funding structures to increase funding for innovative science that can benefit the public’s health.

For such changes as the ones proposed to have impact, additional policies at the NIH need to be enacted to ensure equitable distribution of funds for preliminary studies. One area of concern is the likelihood that opening “new” lines of funding for preliminary studies will be dominated by established investigators, leaving those with less prior grant success or resources struggling to compete [55]. The increasing inequality among investigators and institutions, where the top 10% of investigators receive almost 40% of the total funding and the top 10% of institutions receive over 70% of the total funding [68], is an issue the NIH has attempted to address in the past. This issue requires immediate attention and supersedes those outlined herein, even if no additional funding is allotted for preliminary studies.

## Summary

We acknowledge the complexity of modifying existing funding systems, the challenges and backlash to previous calls for sweeping changes [27], and the unknown ramifications reinvestments in absence of increasing budgets could have on the existing funding scheme. We also recognize without an increase in the NIH budget, any solutions to address the current funding structure within the NIH would result in a zero-sum game. With or without an increase in the NIH budget, the steps outlined herein would ensure the funding of preliminary studies is at the level needed to support innovative and risky-ideas and that is consistent with the translational science frameworks they [NIH] endorse for developing health-related behavioral interventions.

To avoid unnecessary research waste, behavioral scientists proposing new and innovative interventions, and grant reviewers and funding agencies reviewing these interventions, must ensure the preliminary studies proposed and funded are void of known biases that reduce the likelihood of a larger-scale trial being successful [16–18]. We also recognize the proposed issues and solutions may not generalize to other funding agencies within and outside the USA. Nevertheless, as the largest funder of health research in the world [21], the NIH plays an important role in leading by example as communicated by their funding decisions regarding preliminary studies.

In summary, the current disproportionate allocation of funding for larger trials, at the expense of the smaller studies they rely upon, is inconsistent with established behavioral intervention development frameworks and potentially impedes scientific breakthroughs. Adjusting funding allocations to slightly fewer larger-scale trials would more than double the number of existing preliminary studies, increasing the likelihood of accelerating advancements in the behavioral sciences. This type of small reinvestment has the potential to yield large, immediate, and lasting benefits on behavioral science and scientists alike.

## Methods for qualitative quotes

### Data acquisition

We used RePORTER (Research Portfolio Online Reporting Tools, Expenditure and Reports; https://reporter.nih.gov/) to identify all preliminary behavioral intervention studies funded by the NIH. The RePORTER Text Search function was used to identify all projects containing the terms “feasibility” OR “pilot” in the project title, abstract, or terms. Records of all identified studies funded prior to 2019 were downloaded.

### Eligibility

A minimum of two trained research assistants reviewed each NIH Reporter project abstract to determine if it described a pilot/feasibility test of a behavioral intervention. This was defined as studies designed to test the feasibility of a behavioral intervention in human participants and/or provide evidence of a preliminary effect(s) or acceptability/feasibility [16–18]. Consistent with prior studies of preliminary behavioral interventions, abstracts which reported mechanistic studies conducted in laboratories, scale/tool (i.e., exploratory factor analysis), and device development were excluded [17, 18].

### Data synthesis

Using the RePORTER Principal Investigator (PI) search tool, complete NIH funding portfolios were obtained for each PI identified as having at least one NIH-funded pilot/feasibility study through the previously mentioned data acquisition methods. Funding portfolios were downloaded and merged in STATA 16 (STATA Corps., College Station, TX) to create the complete dataset presenting each PIs complete NIH funding portfolio where each pilot/feasibility study was denoted.

A total of 2901 grants, awarded to 2428 unique PIs, were identified as pilot/feasibility studies testing behavioral interventions. Email addresses for each PI were obtained and each PI was sent a link to complete an online Qualtrics survey regarding their perspective on the utility of preliminary studies. The survey was disseminated to all identified PIs beginning November 4th, 2021. Survey reminders were sent out two times with each reminder 7 days apart, for a total of three email distributions. All PIs were allowed to opt-out of the survey and/or survey reminders. The survey remained open for a total of 60 days, closing on January 4th, 2022. All survey questions and distribution methods were approved by the first author’s institutional review board (registration number *Pro00086876*) prior to engaging the first participant. One section of the survey allowed for open-ended free-text responses regarding challenges in the field around designing, conducting, and receiving funding for preliminary studies. The quotes presented represent a sample from those open-ended responses.

### Response rate

A total of 2433 emails addresses were identified across the 2438 PIs represented in the dataset. A total of 308 emails bounced/failed to be delivered, of those, we were able to locate 162 alternative emails and resent the survey to those addresses of which 11 of those failed to be delivered. Two thousand one hundred twenty-five emails successfully reached an inbox and 431 PIs completed at least 75% of the survey for a response rate of 20.3%. Nearly all respondents resided in the USA (98%), and most identified as female (72%). The average age of respondents was 52 (SD 10 years; range 31–80) with an average of 22 post-terminal degree (SD 10 years, range 2–53).

## Supplementary Information

**Additional file 1.** Methods for qualitative quotes.

## Data Availability

NA

## Footnotes

1 See supplemental materials that outline the methods for collecting the quotes.

2 Preliminary studies refer to studies labeled as proof-of-concept, feasibility, exploratory, evidentiary, preliminary efficacy, vanguard, and pilot that are conducted prior to the execution of a larger-scale more well-powered trial.

3 All data presented are publicly available on NIH RePORTER (https://reporter.nih.gov/).

4 Other NIH mechanisms to support larger-scale clinical trials include U01, P grants, and various R01-equivalents (e.g., R23, DP1, DP2) are not included in these analyses as they are awarded less frequently and not available to investigators across all career stages.

5 As noted by Kaplan et al 2017, identifying the amount of NIH funding allotted to behavioral interventions is a complex and imperfect science. For this commentary, to identify behavioral interventions in NIH Reporter we restricted searches to those awarded grants with “intervention” in either the title or abstract. We recognize this is a necessarily imperfect way to capture all behavioral interventions and could result in an over- or under-estimation. In absence of clearer classification of behavioral interventions within the NIH database system, we believe these numbers provide reasonable estimates of behavioral interventions funded from 1985 to 2020.

6 For this analysis we restrict our comparisons for preliminary studies by excluding K awards, since the vast majority of investigators are awarded a career development award once during the early stages of their career, with few K awards issued for mid- and more senior investigators.

7 Defined as an Institute with over $1 billion in annual budget in 2020: NICHD, NIDDK, NINDS, NIGMS, NIA, NHLBI, NIAID, NCI, NIDA

8 Career Development Awards (K Awards) are not considered in these estimates given the mechanism can be received only once during the early career phase (K01, K08, K23, K99) and mid-career and senior research scientist K awards (K02, K05, K18, K22, K24, K26, K43, K76) are not widely awarded.

9 Investigators who are within 10 years of completing postgraduate clinical trialing or their highest advanced research degree
