# Supplementary material for: Fund behavioral science like the frameworks we endorse: the case for increased funding of preliminary studies by the National Institutes of Health
Source: Pilot Feasibility Stud. 2022 Sep 28;8:218. doi: 10.1186/s40814-022-01179-w (PMC9516815; doi:10.1186/s40814-022-01179-w)
Supplement: Supplementary file 1 — Additional file 1. Methods for qualitative quotes. [file 40814_2022_1179_MOESM1_ESM.docx]

**Supplemental Information**

**Methods for Qualitative Quotes**

Data Acquisition. We used RePORTER (Research Portfolio Online Reporting Tools, Expenditure and Reports; <https://reporter.nih.gov/>) to identify all preliminary behavioral intervention studies funded by the NIH. The RePORTER Text Search function was used to identify all projects containing the terms “feasibility” OR “pilot” in the project title, abstract, or terms. Records of all identified studies funded prior to 2019 were downloaded.

Eligibility. A minimum of two trained research assistants reviewed each NIH Reporter project abstract to determine if it described a pilot/feasibility test of a behavioral intervention. This was defined as studies designed to test the feasibility of a behavioral intervention in human participants and/or provide evidence of a preliminary effect(s) or acceptability/feasibility.^16-18^ Consistent with prior studies of preliminary behavioral interventions, abstracts which reported mechanistic studies conducted in laboratories, scale/tool (i.e., exploratory factor analysis), device development were excluded.^17, 18^

Data Synthesis. Using the RePORTER Principal Investigator (PI) search tool, complete NIH funding portfolios were obtained for each PI identified as having at least one NIH-funded pilot/feasibility study through the previously mentioned data acquisition methods. Funding portfolios were downloaded and merged in STATA 16 (STATA Corps, College Station TX) to create the complete dataset presenting each PIs complete NIH funding portfolio where each pilot/feasibility study was denoted.

A total of 2,901 grants, awarded to 2,428 unique PIs, were identified as pilot/feasibility studies testing behavioral interventions. Email addresses for each PI were obtained and each PI was sent a link to complete an online Qualtrics survey regarding their perspective on the utility of preliminary studies. The survey was disseminated to all identified PIs beginning November 4^th^, 2021. Survey reminders were sent out two times with each reminder seven days apart, for a total of three email distributions. All PIs were allowed to opt-out of the survey and/or survey reminders. The survey remained open for a total of 60 days, closing on January 4^th^, 2022. All survey questions and distribution methods were approved by the first author’s institutional review board (registration number *Pro00086876*) prior to engaging the first participant. One section of the survey allowed for open-ended free-text responses. The quotes presented represent a sample from the open-ended responses.

Response Rate. A total of 2,433 emails addresses were identified across the 2,438 PIs represented in the dataset. A total of 308 emails bounced/failed to be delivered, of those, we were able to locate 162 alternative emails and resent the survey to those addresses of which 11 of those failed to be delivered. 2,125 emails successfully reached an inbox and 431 PIs completed at least 75% of the survey for a response rate of 20.3%. Nearly all respondents resided in the United States (98%), and most identified as female (72%). The average age of respondents was 52 (SD 10 years; range 31-80) with an average of 22 post-terminal degree (SD10 years, range 2-53).
